# Kinematic demands of FIFA’s EPTS validation circuit compared to a sport-specific alternative

**DOI:** 10.1038/s41598-025-31587-w

**Published:** 2025-12-09

**Authors:** Rafael Luiz Martins Monteiro, Paulo Roberto Pereira Santiago, Patrick Blauberger, Guilherme de Sousa Pinheiro, Tiago Guedes Russomanno, Martin Lames

**Affiliations:** 1 Program in Rehabilitation and Functional Performance, Ribeirão Preto Medical School, University of São Paulo, Ribeirão Preto, 14049-900 Brazil; 2 Biomechanics and Motor Control Laboratory, School of Physical Education and Sports of Ribeirão Preto, University of São Paulo, Ribeirão Preto, 14040- 907 Brazil; 3 Chair of Performance Analysis and Sports Informatics, Technical University of Munich, 80992 Munich, Germany; 4 Faculty of Physical Education, University of Brasilia, Brasilia, 70910-900 Brazil

**Keywords:** Soccer, Football, Global positioning system, Position data, Motion capture, Biological physics, Biological techniques, Mathematics and computing

## Abstract

This study compared the kinematic demands of FIFA’s Electronic Performance and Tracking Systems (EPTS) validation circuit with an alternative sport-specific circuit (SSC). The aim was to determine which circuit presents high-intensity demands known to challenge EPTS accuracy. Four amateur soccer players (age: 29.4 ± 5.9 years) performed both circuits using global positioning system (GPS). Speed, acceleration and change of direction (COD) was divided into zones and compared between the circuits. Results showed that the SSC resulted in greater distance, time and entrances in speed zones 3 and 4, as well as in moderate and high acceleration/deceleration zones. SSC also exhibited more time and entries in high-speed COD zones. FIFA’s circuit demonstrated higher peak speed and more time in speed zone 5. Differences in COD between circuits reflect the intensity and type of exercises performed, with the SSC including more sprints and rapid direction changes. In conclusion, the SSC presents more kinematic demands in high-intensity situations critical for EPTS validation, whereas the FIFA circuit demonstrates less challenging movement patterns. FIFA could improve the validation protocol by updating the circuit to include more demanding sport-specific movements thus increasing the evaluated devices validity in high-intensity situations which are of very high interest for sports.

## Introduction

In 2019 the *Fédération Internationale de Football Association* (FIFA) extended its Quality Programme for Electronic Performance and Tracking Systems (EPTS) beyond only safety tests to include methods to quantify the accuracies of optical and wearable devices^1^. There are three kinds of EPTS that can be evaluated in the FIFA Quality Programme: Optical Tracking Systems (OTS), Local Positioning Systems (LPS) and Global Positioning Systems (GPS)/ Global navigation satellite systems (GNSS). A review^2^ showed that different criterion methods have been used for EPTS validation. The most common involve: (1) Circuits with predefined movements and known distances: this approach evaluates distance accuracy^3–7^; (2) Timing gates: used to assess average speed^3,4,6–9^; and (3) Radar/laser-based systems: measure instantaneous running speed^10–12^. However, these methods have limitations. Linke et al. (2018), Ogris et al. (2012) and Luteberget & Gilgien (2020)^2,13,14^ highlight the need of a comparison involving a two- or three-dimensional reference system with established error estimates, which would constitute ideal validation conditions.

**Fig. 1:**
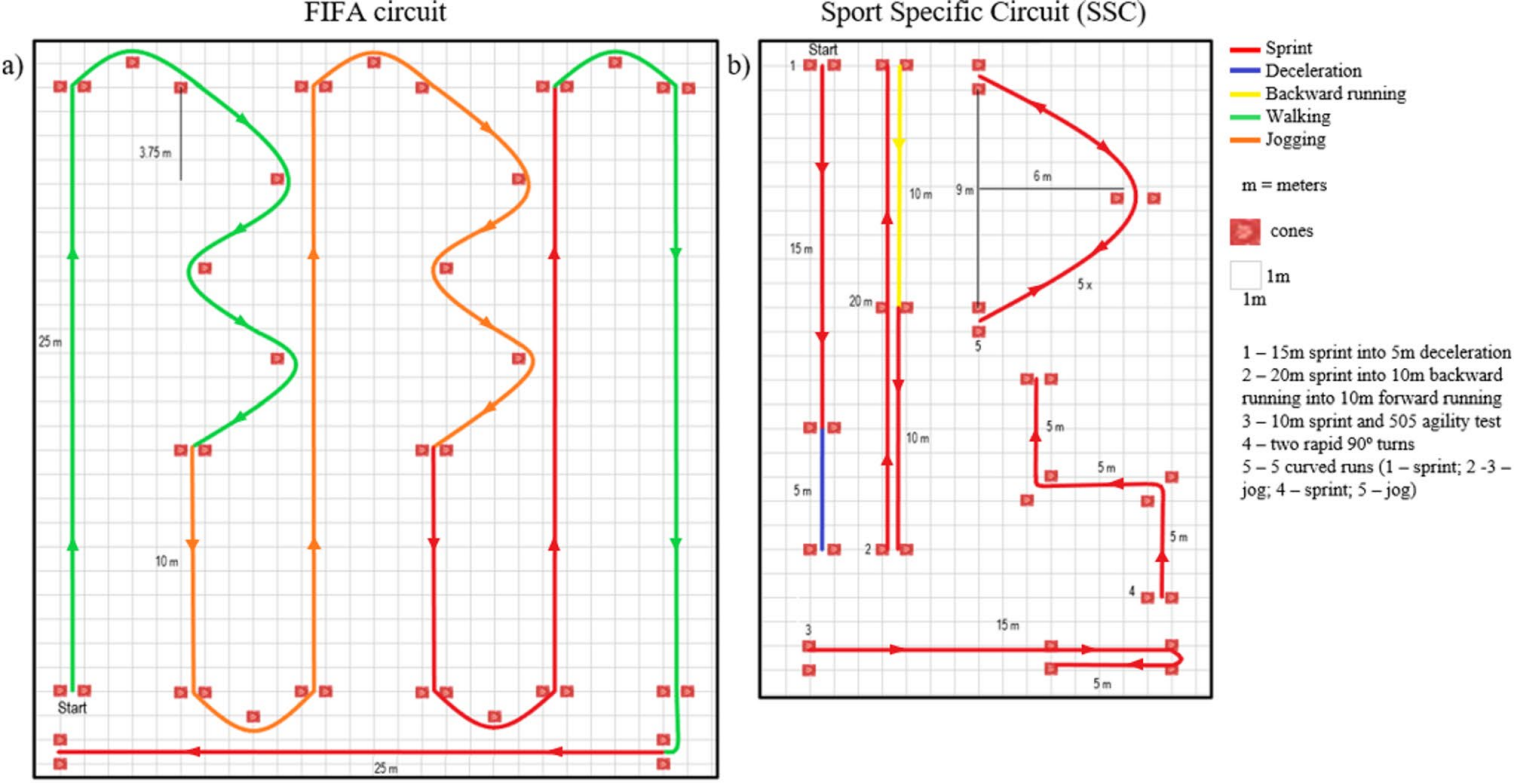
Experimental setup of the circuits. (**a**) FIFA circuit; (**b**) Sport specific circuit. The direction of the exercises is indicated by colored triangles.

**Fig. 2:**
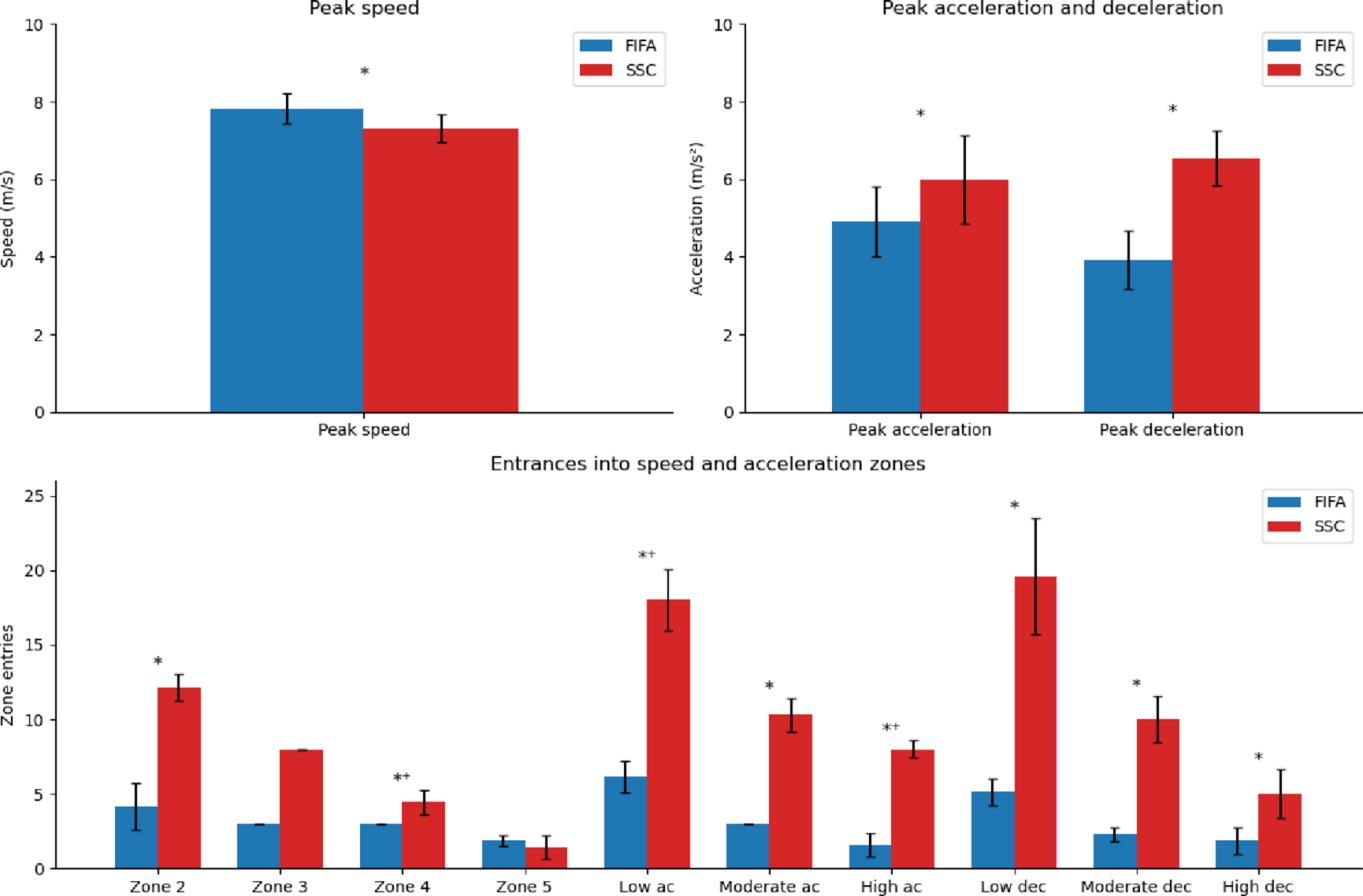
Peak speed, acceleration and deceleration and entrances into speed and acceleration/deceleration zones. m, meters; s, seconds; FIFA, FIFA circuit; SSC, Sport specific circuit; ac, acceleration; dec, deceleration; *p < 0.05; ⁺did not pass the normality test.

The FIFA testing protocol needs to be conducted in a football stadium respecting the official FIFA regulations for pitch dimensions. The accuracy evaluation is done with the positional, speed and acceleration data comparison with gold standards motion capture systems: (1) Vicon for testing specific football movements in a certain area of the pitch, (2) laser gun to measure high speed sprints, and (3) a total station for a pitch survey to ensure that the accuracy of the manufacturer’s system can be assessed and prepared for real life game scenarios^15,16^. The FIFA testing protocol for EPTS validation includes: (1) a circuit with self-paced walking, self-paced jogging, maximal accelerations and change of directions (Fig. 1a), (2) 2 v 2 and 5 v 5 small-sided games, (3) Series of maximal sprints, and (4) moving around the pitch staying within the pitch lines^15–17^.

Considering that positional data obtained by EPTS is used daily by soccer clubs for extracting important informations, such as tactical performance analysis^18,19^ and monitoring external (locomotor) load^18,20,21^, it is essential that it has a proper validation. The high-intensity efforts need to be covered in the design of a circuit for EPTS validation. Studies have shown that a Sport Specific Circuit (SSC) captured the tested devices inaccuracies in specific exercises involving sprints, abrupt acceleration/decelerations, and sequences of directional changes^2,22,23^. These movements not only represent realistic sport demands that are present in key moments of a soccer match but also specifically target the scenarios where EPTS accuracy is most compromised. Match-analysis studies have shown that linear sprints, followed by decelerations and turns are the most common movements preceding goals^24,25^. Oliva-Lozano & Muyor (2022)^17^ pointed important limitations on the FIFA’s EPTS validation protocol by analyzing public reports from devices that have already been validated by FIFA. They identified that not all EPTSs were tested for the same drills and some of them didn’t present results for high-speed zones (20–25 km/h or above 25 km/h). Some devices were tested with under-13 soccer players, who may not be capable of reaching these high-speed zones, during small-sided soccer games and the FIFA circuit.

A well-designed circuit for EPTS validation should incorporate movements relevant to team sports, with a particular focus on scenarios that pose challenges for position tracking devices^23^. The scientific literature has demonstrated that EPTS accuracy tends to decrease with increasing speed and during acceleration/deceleration^2,23,26,27^. The intensity and kind of exercises appear to have critical influence on the error’s magnitude. In LPS validation, fast changes of direction (COD) (90° turns) can lead to a significant increase of the spatial error when compared to other exercises^2^. The peak speed and acceleration/deceleration have also presented more errors in zig-zag jogging, squat jump and change of direction exercises^23^. Regarding OTS validation the 505-agility test presented higher spatial errors^2^. GPS seems to be capable of accurately measuring distances with low and moderate speed but tends to present more errors in exercises with high-speed direction changes^2,28,29^. Regarding the GPS speed measuring accuracy this device seems to present more errors in linear and frequent change of direction high speed running^29^.

In summary, the design philosophy of the circuit that FIFA uses for EPTS validation (Fig. 1) doesn’t seem to be a thorough check of the systems’ capabilities to meet the information demands of sports practice as critical but interesting situations for position tracking devices are omitted or under-represented. The small-sided games (SSG) from FIFA protocol don’t seem to present this critical situation either, considering that SSG have presented minor challenging kinematic demands when compared to predefined circuits in LPS validation for example^23^. While previous studies have focused on comparing EPTS accuracy to gold standard systems, the novelty of this study is the systematic evaluation of testing protocols to enhance the EPTS validation process. Considering that predefined circuits for EPTS validation should expose the testing devices to “worst case scenarios” they may face in practice, the present study aimed to compare the kinematic demands of FIFA circuit and an alternative Sport Specific Circuit (SSC)^2,22,23^, pointing out what circuit presented more critical situations that may increase the error’s magnitude. Evaluating testing protocols is essential for refining the process of EPTS validation. The authors’ hypothesis was that the SSC would present higher kinematic demands specifically in high-speed, high-acceleration/deceleration, and rapid change-of-direction situations and, consequently, generate more data in scenarios where EPTS devices tend to show elevated error magnitudes.

## Methods

### Participants

Four adult male amateur soccer players with regular sports practice (age: 29.4 ± 5.9 years, height: 1.75 ± 0.03 m, body mass: 69 ± 5.23 kg) participated in the study. To be included in the sample, participants had to reach at least 25 km/h in a 40-m sprint. This speed threshold was introduced as inclusion criterion to make sure that high-intensities were potentially realized, especially the higher speed zone (> 25 km/h) from FIFA’s EPTS validation protocol^16^. While the low number of participants is a limitation, the study’s focus on comparing circuits’ kinematic characteristics means that the results primarily reflect the nature of exercises in each circuit. Nevertheless, a larger sample size would be recommended for future EPTS validation studies to better assess measurement error and increase statistical power. This study fulfilled the Technical University of Munich’s ethical standards which include alignment to the Declaration of Helsinki recommendations and the requirement of obtaining informed consent from all participants, who were made aware that all data would be anonymized. The data collection was part of the practical activities from the Workshop FAPESP-BAYLAT: “large volume—high precision systems for position detection in sports”, process number #19/22262-3, and had its experimental protocol approved by the organizing committee formed by an official agreement between the School of Physical Education and Sport of Ribeirão Preto, University of São Paulo and Technical University of Munich.

### Instruments

Two circuits were set up on a soccer pitch using cones (Fig. 1). One of the circuits was proposed by International Federation of Football Associations (FIFA)^15,17,30^ for validating EPTS and the other is a sport-specific circuit (SSC) that has been used in other studies with this same purpose and includes realistic soccer exercises specifically designed to challenge tracking accuracy through sprints, rapid accelerations/decelerations, and directional changes^2,22,23^. The athletes’ x–y positions, speed, and acceleration data were collected using 4 commercially available GNSS (global navigation satellite systems) wearable devices (Kinexon GPS Pro powered by Fitogether: OHCOACH cell X, 10 Hz GPS, GLONASS, Galileo, Beidou with QZSS and SBAS; Accelerometer 100 Hz ± 16 g Tri-axial; Gyroscope 100 Hz ± 2000 dps Tri-axial; Magnetometer 100 Hz ± 50 gauss Tri-axial; Dimension 45 mm x 76 mm x 18.3 mm; Weight 51 g—Kinexon Sports & Media GmbH, Munich, Germany). This GNSS system holds a FIFA Quality and Basic certification. Following manufacture’s recommendation, the data collection was done in an open-sky soccer field and after the GPS initialization the data collection only started when the Kinexon system signalized the devices where synchronized. Signals from 32 satellites were used for data acquisition. Each athlete wore a sport vest with the GPS device attached to the back and soccer shoes with cleats suitable for high agility on a lawn surface.

### Experimental procedures

The data collection was carried out on an open-sky soccer field of the Technical University of Munich. Before starting the circuits, participants received verbal explanations about the tests and a demonstration on how each circuit should be performed. They also had free time to warm up. Participants performed each circuit twice and had self-selected resting times. The order of the circuits was randomized for the first trial. For example: the subject could start by performing the FIFA circuit, then the SSC circuit twice and finally, the FIFA circuit again. The sequence was counterbalanced across participants to minimize order effects.

During the data collection there was always a research assistant guiding the participants through the circuits. The assistants were pointing out what was the next step of the circuit and giving verbal instructions and encouragement. Each participant also performed a 40-m sprint before starting the first circuit and after the last with instantaneous maximum speed feedback to check if the athlete could reach 25 km/h in all trials. One of the 4 participants ran the circuits only once, resulting in 7 runs in the FIFA circuit and 7 in the SSC for the analysis.

### Data processing

For data processing each run was cut individually based on the local positioning variables and the doppler speed curve of each GPS. For general analysis the following parameters were calculated for each trial: total distance covered, peak speed, peak acceleration, peak deceleration, and total time to perform each circuit^20^. The speed was divided into 5 different zones and per zone the following parameters were calculated: distance covered, time spent and how many times the participant reached each zone. The speed thresholds were: zone 1—walking (< 6 km h ^− 1^), zone 2—jogging (≥ 6 to < 15 km h^− 1^), zone 3—running (≥ 15 to < 20 km h^− 1^), zone 4—high-speed running (≥ 20 to < 25 km h^− 1^), zone 5—sprinting (≥ 25 km h^− 1^)^23^.

The acceleration and deceleration were also divided into thresholds and distance covered, time spent and how many times the participant reached each zone were calculated. The acceleration/deceleration thresholds used were the same FIFA proposes for EPTS valdation: very low (< 1 m s^−2^), low (≥ 1 to < 2 m·s^−2^), moderate (≥ 2 to < 3 m s^−2^) and high (≥ 3 m s^−2^)^16,31^. Following the FIFA’s protocol for noise treatment in EPTS data, the Doppler speed provided by the GNSS wearable devices manufacturer and the x–y position data was filtered using a 4th order low-pass Butterworth filter with cutoffs frequency of 1 Hz. The acceleration and distance to realize each circuit were calculated using the difference in filtered instant speed at each GPS time stamp^16^.

The Change of Direction (COD) was calculated using the x–y provided by the GNSS. This measure is dependent on the device sampling rate with prior studies showing that 10 Hz devices overcome the limitations presented by low sample rate (1–5 Hz), especially in COD situations. Despite these advances, 10 Hz GPS devices are still prone to errors in high intensity COD situations, especially when compared to higher frequencies tracking systems, such as 20 Hz LPS^6^. The angular displacement was calculated from the filtered data and then the angular speed for each GPS time stamp, which represents the COD. The angular acceleration was calculated using the COD at each GPS time stamp. The COD was divided into thresholds and calculated the time spent and how many times the participant reached each zone across the different speed zones. This analysis allows to determine in which speed zone each COD zone was reached and for how long. Higher COD zones in higher speed zones represents a scenario that EPTS tends to present more errors^2,23,28,29^. The COD thresholds used were: very low ( 30°·s⁻¹), low (≥ 30°·s⁻¹ to 60°·s⁻¹), moderate (≥ 60°·s⁻¹ to 90°·s⁻¹), and high (≥ 90°·s⁻¹).

### Statistical analysis

For statistical analysis the mean and standard deviation of the trials in FIFA and SSC circuits were calculated. The Shapiro–Wilk was used to indicate if the data had normal distribution. For comparing the results between the two circuits a Paired Student’s t test was used for the variables that presented normal distribution and for non-parametric data the Wilcoxon test was used. Cohen’s d (d) was used to report the effect size of the presented variables (0.2 small, 0.5 medium, > 0.8 large)^32^. Large Cohen’s d values (d > 1.0) reflect the combination of a pronounced difference in mean values and low variability within a small sample size. While statistically correct, these values should be interpreted with caution and considering the specific conditions of the present study. The statistical analysis was conducted in the jamovi project (2024). jamovi (Version 2.3.28) [Computer Software]. Retrieved from https://www.jamovi.org.

## Results

All participants reached zone 5 in the sprints of 40 m, before and after running the circuits, which shows that they were able to reach this speed zones during the FIFA and SSC circuits (Table 1).

**Table 1:** Speed reached by each participant in the 40 m sprint before and after running the circuits in meters per second and Kilometers per hour.

Player	40 m sprint speed (m/s) (km/h)	
	First trial	Second trial
1	7.96 (28.7)	7.85 (28.3)
2	8.51 (30.6)	8.58 (30.9)
3	8.29 (29.8)	8.30 (29.9)
4	7.82 (28.1)	7.53 (27.1)

Comparing total demands between circuits, the SSC required participants to cover greater distances and spend more time completing the protocol, with marked differences in high-intensity speed and acceleration/deceleration zones. Specifically, higher values were observed for total distance (*p* < 0.001, d = 2.3) and total time to complete the circuit (*p* = 0.002, d = 2). In the speed zones, the SSC circuit presented greater distances in zones 2 (*p* = 0.002, d = 2), 3 (*p* < 0.001, d = 21.2), and 4 (*p* = 0.001, d = 2.2), as well as longer time spent in these same zones: zone 2 (*p* < 0.001, d = 3), 3 (*p* < 0.001, d = 24.4), and 4 (*p* = 0.001, d = 2.16). For the acceleration/deceleration zones, the SSC circuit also showed greater distances in the low (*p* < 0.001, d = 2.3), moderate (*p* = 0.02, d = 6.6), and high (*p* < 0.001, d = 4.3) zones, along with longer time in these same zones: low (*p* < 0.001, d = 5.1), moderate (*p* < 0.001, d = 6.9), and high (*p* < 0.001, d = 5). In contrast, the FIFA circuit resulted in greater distance covered in speed zones 1 (*p* = 0.004, d = 1.7) and 5 (*p* = 0.001, d = 2.2), as well as in the very low acceleration zone (*p* < 0.001, d = 10.1), and a longer time in speed zone 5 (*p* = 0.001, d = 2.1) (Table 2).

**Table 2:** Kinematic descriptive variables of time and distance in total and each speed and acceleration/deceleration zones in the FIFA and SSC circuits.

	Zones	Distance (m)		Time (s)	
		FIFA	SSC	FIFA	SSC
Speed	Total	228.4 ± 6.1*	279.3 ± 24.2*	115.5 ± 9.7*	142.4 ± 15.6*
	Zone 1	89.8 ± 8.2*	65 ± 18.6*	72.6 ± 10.4	77.8 ± 18.2
	Zone 2	77.2 ± 6.3*	104.6 ± 5.2*	32.9 ± 1.8*	43.8 ± 3.1*
	Zone 3	15 ± 2.5*	60.2 ± 1.5*	3.1 ± 0.5*	12.5 ± 0.5*
	Zone 4	24.1 ± 3.5*	40.3 ± 6.6*	3.9 ± 0.6*	6.5 ± 1.1*
	Zone 5	22.41 ± 6*	9.1 ± 7.2*	3 ± 0.8*	1.3 ± 1*
Acceleration/deceleration	Very low	184.3 ± 5.9 *	166.3 ± 14.7*	104.4 ± 10	105.69 ± 13.9
	Low	24.5 ± 2.8*	56.1 ± 13.6*	6.1 ± 1.1*	18.7 ± 2.8*
	Moderate	13.3 ± 2.6*^+^	33.6 ± 4.5*^+^	3.13 ± 0.7*	9.5 ± 1.4*
	High	6.4 ± 2.8*	23.3 ± 4.9*	1.7 ± 0.7*	8.4 ± 1.5*

m, meters; s, seconds; FIFA, FIFA circuit; SSC, Sport Specific circuit; *p < .05; ⁺did not pass the normality test.

The SSC resulted in higher peak acceleration (6 ± 1.1 vs. 4.9 ± 0.9 m/s^2^, *p* < 0.001, d = 1.2) and peak deceleration (6.5 ± 0.7 vs. 3.92 ± 0.7 m/s^2^, *p* < 0.001, d = 5.7) compared to the FIFA circuit. In contrast, the FIFA circuit showed higher peak speed (7.82 ± 0.4 vs. 7.3 ± 0.4 m/s, *p* = 0.01, d = 1.4). Regarding the number of zone entrances, the SSC circuit demonstrated more entrances into speed zones 2 (12.1 ± 0.9 vs. 4.1 ± 1.6, *p* < 0.001, d = 4.9) and 4 (4.43 ± 0.8 vs. 3, *p* = 0.02, d = 1.8), as well as greater entrances into acceleration zones: low (18 ± 2.1 vs. 6.1 ± 1.1, *p* < .001, d = 5.2), moderate (10.3 ± 1.1 vs. 3, *p* = 0.02, d = 6.5), and high (8 ± 0.6 vs. 1.6 ± 0.8, *p* < 0.001, d = 12). Similarly, the SSC also presented higher entries into deceleration zones: low (19.6 ± 3.9 vs. 5.1 ± 0.9, *p* < 0.001, d = 3.8), moderate (10 ± 1.5 vs. 2.3 ± 0.5, *p* < 0.001, d = 5.6), and high (5 ± 1.6 vs. 1.9 ± 0.9, *p* < 0.001, d = 1.9) (Fig. 2).

Regarding the time spent in COD zones across different speed zones, the SSC circuit resulted in higher time in very low COD within speed zones 2 (*p* < 0.001, d = 3.5), 3 (*p* < 0.001, d = 3.2), and 4 (*p* < 0.001, d = 2.9); in low COD within speed zones 1 (*p* < 0.001, d = 3.9) and 3 (*p* = 0.005, d = 1.6); in moderate COD within speed zones 1 (*p* < 0.001, d = 4.3) and 3 (*p* < 0.001, d = 3.2); and in high COD within speed zone 1 (*p* < 0.001, d = 2.3). In contrast, the FIFA circuit showed higher time in very low COD within speed zones 1 (*p* = 0.03, d = 1.1) and 5 (*p* = 0.001, d = 2.1), in low COD within speed zone 4 (*p* = 0.03, d = 1.1), and in high COD within speed zone 2 (*p* = 0.02, d = 1.2) (Table 3).

**Table 3:** Distribution of time spent in change of direction zones across different speed zones.

	Zones	Time in change of direction zones (s)							
		Very low		Low		Moderate		High	
		FIFA	SSC	FIFA	SSC	FIFA	SSC	FIFA	SSC
Speed	Zone 1	60.9 ± 8.8*	46.9 ± 13.6*	6.4 ± 1.7*	14.5 ± 2.5*	3 ± 0.9*	6.4 ± 1*	2.3 ± 0.5*	9.9 ± 3.3*
	Zone 2	20.4 ± 2.2*	33.5 ± 3.8*	9.4 ± 0.9	8.5 ± 1.3	2.2 ± 0.8	1.6 ± 0.8	0.9 ± 0.7*	0.2 ± 0.2*
	Zone 3	1.3 ± 0.7*	7.6 ± 0.5*	1.4 ± 0.3*	2.3 ± 0.4 *	0.4 ± 0.2*	1.7 ± 0.3*	-	0.9 ± 0.3
	Zone 4	3.2 ± 0.8*	6.3 ± 0.8*	0.7 ± 0.3*	0.1 ± 0.4*	-	0.04 ± 0.1	-	-
	Zone 5	3 ± 0.8*	1.3 ± 1*	-	-	-	-	-	-

s, seconds; FIFA, FIFA circuit; SSC, Sport specific circuit; *p < 0.05.

**Fig. 3:**
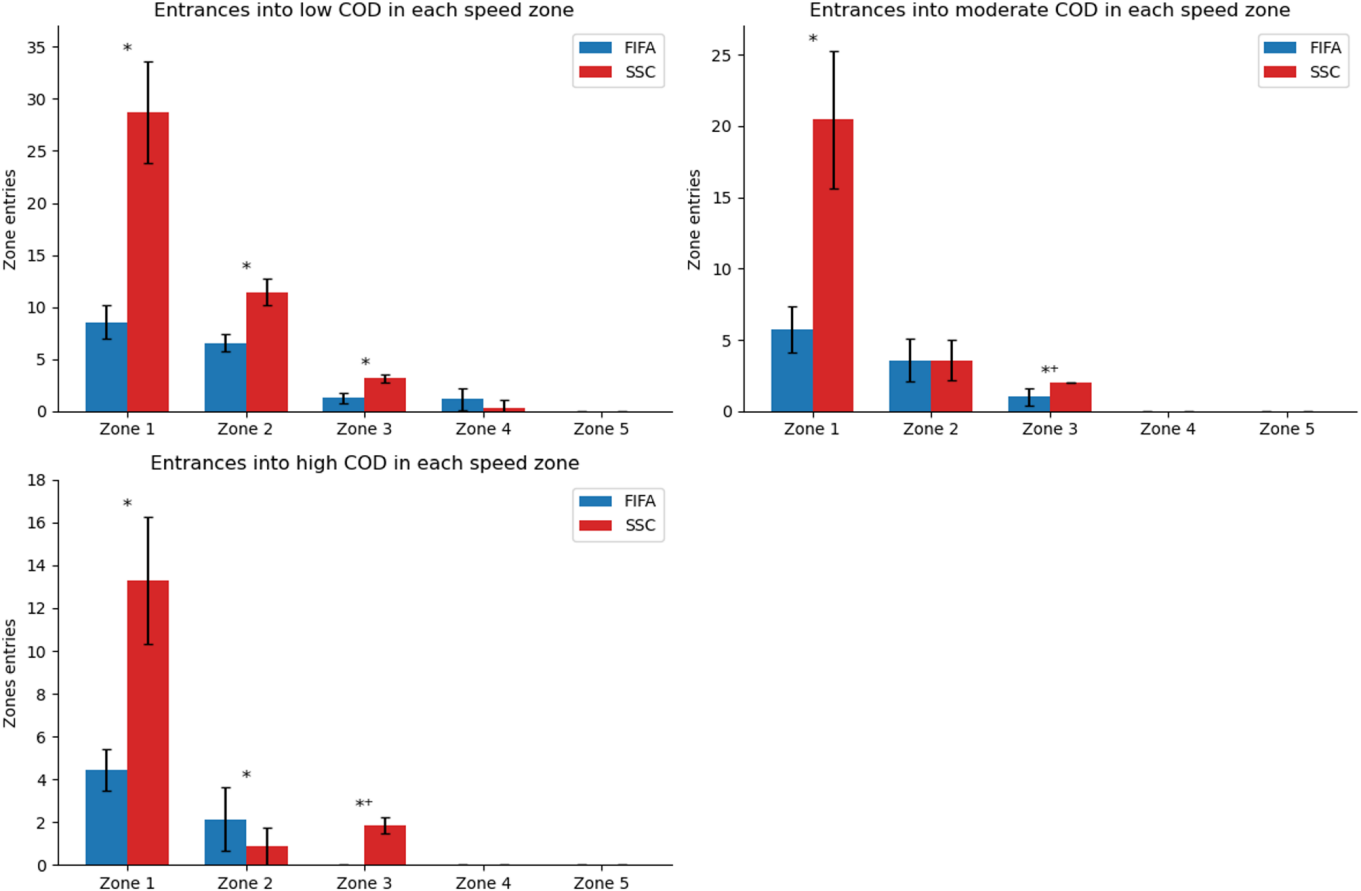
Entrances into change of direction zones across different speed zones. FIFA, FIFA circuit; SSC, Sport specific circuit; COD, Change of direction; *p < 0.05; ⁺did not pass the normality test.

**Fig. 4:**
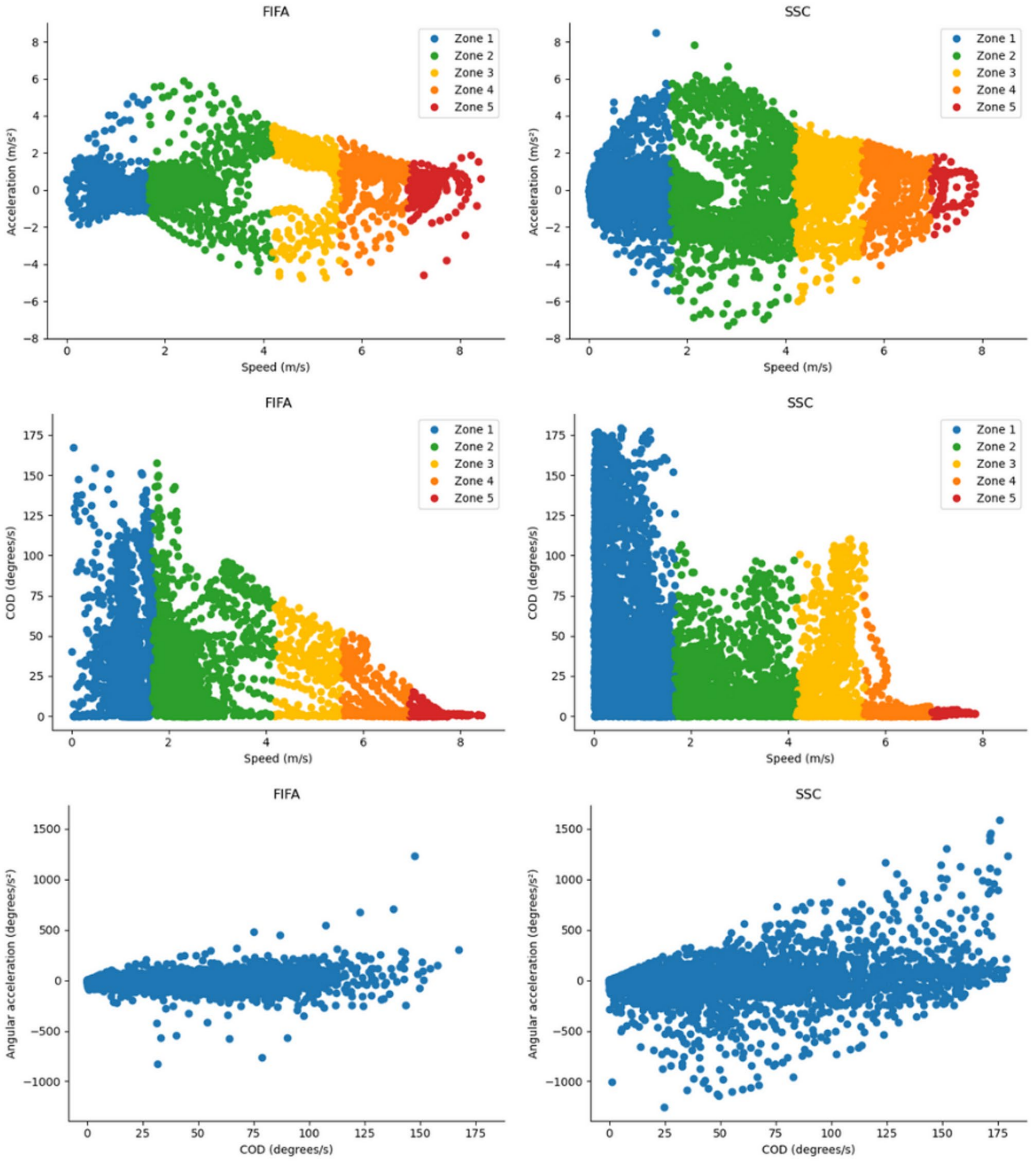
Distribution of speed with acceleration and change of direction with data divided by speed zones and distribution of angular acceleration and change of direction. m, meters; s, seconds; FIFA, FIFA circuit; SSC, Sport specific circuit; COD, Change of direction. The COD were filtered for a maximum of 180°/s for visualization. The COD above this filter occurred only in speed zone 1.

Regarding the number of entrances into COD zones across different speed zones, the SSC circuit showed a higher number of entrances in low COD within speed zones 1 (28.7 ± 4.9 vs. 8.6 ± 1.6, *p* < 0.001, d = 4.7), 2 (11.4 ± 1.3 vs. 6.6 ± 0.8, *p* < 0.001, d = 3.1), and 3 (3.1 ± 0.4 vs. 1.3 ± 0.5, *p* < 0.001, d = 2.7); in moderate COD within speed zones 1 (20.4 ± 4.8 vs. 5.7 ± 1.6, *p* < 0.001, d = 3.2) and 3 (2 vs. 1 ± 0.6, *p* = 0.03, d = 1.7); and in high COD within speed zones 1 (13.3 ± 3 vs. 4.4 ± 1, *p* < 0.001, d = 2.6) and 3 (1.9 ± 0.4 vs. 0, *p* = 0.02, d = 4.9). In contrast, the FIFA circuit resulted in a higher number of entrances in high COD within speed zone 2 (2.1 ± 1.5 vs. 0.9 ± 0.9, *p* = 0.02, d = 1.2) (Fig. 3). The distributions of speed, acceleration, COD, and angular acceleration across both circuits are illustrated in Fig. 4.

## Discussion

The present study aimed to compare the kinematic demands of FIFA circuit and an alternative SSC. Our investigation has demonstrated that the SSC circuit is slightly longer, with a difference of approximately 50.9 m (22.28%) and takes more time to be performed, about 26.9 s (23.29%). Considering that EPTS tends to present more errors in higher speed zones, higher acceleration/deceleration and high-speed COD^2,23,26–29^, the results clearly demonstrate that the SSC circuit presents more critical situations for position tracking devices and consequently representing a circuit with better sport specific movements for a complete EPTS validation.

These critical situations can be seen in the SSC by the higher distance and time spent in higher speed zones 3 and 4 and acceleration/deceleration zones moderate and high (Table 2). It also appears by analyzing the number of entrances in this same speed and acceleration/deceleration zones and peak acceleration and deceleration (Fig. 2). These results presented large effect sizes which implies significant practical differences and can be explained by the higher number of sprints in the SSC circuit (6) when compared to the FIFA circuit (2) which ends up resulting in more high-intensity accelerations and decelerations that are very common during soccer games and preceding goal situations^25,33^. The importance of a proper validation of high speed/acceleration situations is of very high interest considering that distance covered in high-speed running and sprints has historically increased in male and female soccer^34^. Therefore, the FIFA circuit presented higher peak speed, distance, and time in speed zone 5. This happened due to the two 25 m sprints that are longer than the sprints in SSC circuit.

Regarding the COD it is important to highlight that higher COD usually occurs in very low speeds. This phenomenon is attributed to the fact that when the COD surpasses a 90° angle, the individual in question needs to execute a return maneuver, necessitating to almost stop for changing direction. The SSC presented more time and entrances in very low, low, moderate, and high COD zones in speed zone 3, that is a running speed threshold (Table 3; Fig. 3). The greater data in zone 3 and higher COD zones can be clearly seen in Fig. 4. The SSC also demonstrated more time in very low COD in zone 4. The FIFA circuit presented higher time in very low COD in speed zone 5 and low COD in speed zone 4, probably because of the more time the circuit presented in speed zone 5 and the sprinting turn (Fig. 1). These differences in the COD across speed zones show that in general the SSC presented more high-speed COD.

These COD results can be explained by the intensity and the kind of exercises in each circuit. The 505-agility test, two rapid 90° turns and the curved sprints and joggings present in the SSC contributed to generating more high-speed COD results. These three exercises are interesting for EPTS validation since they are specifically challenging for the different kinds of systems and turns are very common movements preceding goals^25^. LPS has demonstrated more errors in rapid 90º turns and zig-zag compared to other exercises^2,23^, OTS has presented more spatial errors in the 505-agility test^2^ and GPS seems to present more errors in high-speed direction changes^2,28,29^. The error may arise from different technological limitations, for instance, GNSS/GPS systems may exhibit positional lag during rapid changes of direction, while both OTS and LPS systems might struggle to accurately filter data during the abrupt velocity changes inherent in high-acceleration and deceleration movements. Future studies could contribute to EPTS development by exploring why these tracking devices present errors in these specific combinations of intensity and kind of exercises. Regarding the COD in lower speed zones (1 and 2) both circuits alternated in which presented more time spent and occurrences. It is closely linked with the walking characteristics of each circuit (Fig. 1).

All participants satisfied the inclusion criterion of achieving speed zone 5 in the 40-meter sprint, both before and after completing the circuit activities (Table 1). This criterion was used to address the limitation of using amateur players and ensures that participants possess the capacity to reach high-speed zones during testing, which is crucial as previous research suggests these zones are particularly prone to EPTS inaccuracies^2,23,26,27^. Future FIFA’s EPTS validation protocols and other studies in this area should consider implementing such an inclusion criterion because it offers a practical approach to avoid testing protocols where high-speed zones are not attained^17^. This ensures the relevance of the testing data to real-world soccer scenarios where professional players frequently reach high speeds^33^. EPTS validation protocols can also consider using a minimum amount of data in each speed zone as requirement for accuracy evaluation considering the minimum distance or time in that zone accordingly to the validation protocol goals.

Limitations of the present study also include the low number of participants and the use of GNSS/GPS devices which are likely to present errors. However, these limitations do not compromise the validity of the findings. The GPS device employed in this study holds both FIFA Quality and Basic certifications. While GNSS/GPS data are prone to errors due to environmental conditions specific to the testing session, such inaccuracies are particularly relevant for an effective validation of EPTS. Moreover, the kinematic demands obtained align with expectations because they reflect the kind of sport-specific exercises of each circuit.

When choosing a circuit for EPTS validation, it is crucial to include sport-specific movements that can create challenging situations for EPTS accuracy^23^. This is even more desirable considering that these challenging situations are high-intensity situations which makes them most interesting to athletic coaches for peak load assessment, has historically increased in soccer and precedes decisive moments in soccer matches^24,25,34^. Correctly evaluating the devices across different movement patterns and intensity zones is essential for providing reliable data to soccer clubs and for the continuous improvement of tracking systems. FIFA’s EPTS validation protocol is essential for reliable data assurance and beyond the circuit also includes other components such as small-sided games and maximal sprints. FIFA embraces the challenge of optimally testing different tracking devices with a single protocol which is why they are continuously updating and improving the EPTS validation protocol. Through a kinematic demands analysis it was possible to describe and compare the data SSC and FIFA circuits provide for the EPTS validation. The circuit used by FIFA seems not to be challenging for the EPTS and doesn’t cover all the critical situations in which the errors magnitude tends to rise. The FIFA circuit lacks diversity in its exercise repertoire, particularly in movements that involve high acceleration/deceleration and rapid changes of direction.

FIFA should consider updating the circuit for EPTS validation, not necessarily using the SSC compared in the present study but mixing the circuits strengths into a new one. Since FIFA’s circuit aims to evaluate different types of EPTS it should address all the specific limitations of the devices. From SSC exercises FIFA should consider adding sport-specific movements such as rapid 90 degrees COD, 505 agility tests, acceleration followed by deceleration and curved runs to be sure that the errors in situations with high-speed COD and -acceleration and -deceleration are being evaluated. From the present FIFA’s circuit, the long 25 m sprint could be used in a new circuit, for example making the first and second sprints from the SSC longer.

## Conclusion

The SSC presented more kinematic demands in which the EPTS tends to present more errors like exercises with high speeds, high acceleration/deceleration, and high-speed COD. These critical situations can be seen in the SSC by the higher distance, time spent and entrances in higher speed zones 3 and 4 and acceleration/deceleration zones moderate and high. The variables in the FIFA circuit that may have more potential for EPTS accuracy errors are associated with the two 25-m sprints, which are longer than those in the SSC. FIFA’s circuit used in the quality programme for EPTS validation can be updated to provide an evaluation that covers more sport-specific relevant movements that tends to increase the magnitude of errors on the testing devices. FIFA should consider adding rapid 90 degrees COD, 505 agility tests and curved runs to be sure that the errors in sports relevant situations such as high-speed, rapid change of direction and high-intensity acceleration/deceleration are being evaluated. Additionally, adding a criterion to ensure participants can perform activities at high speeds is essential to guarantee accuracy validation in all speed zones. These changes could contribute to a more effective EPTS validation process. To develop better validation procedures, it is essential to evaluate and compare existing protocols. Future studies could compare error measurements from different testing protocols against gold standard systems, such as Vicon, enabling direct assessment of which protocol configurations best reveal EPTS limitations.

## Data Availability

The datasets generated and analyzed during the current study and the code for the data analysis pipeline performed are available on figshare (10.6084/m9.figshare.27018445).
